# Considerations for Future Research and Methodological Clarifications on Smoking Behavior Change and Heart Failure Risk in Patients With Type 2 Diabetes

**DOI:** 10.2196/60713

**Published:** 2024-11-12

**Authors:** Jana Malinovská, Juraj Michalec, Jan Brož

**Affiliations:** 1 Department of Internal Medicine, Second Faculty of Medicine, Charles University, V Úvalu 84, Prague, 15000, Czech Republic, 420 224434001, 420 224434019

**Keywords:** type 2 diabetes, smoking, heart failure, cardiovascular disease, smoking cessation

We read with great interest the article by Yoo et al [1], titled “Smoking Behavior Change and the Risk of Heart Failure in Patients With Type 2 Diabetes: Nationwide Retrospective Cohort Study,” published in this journal (*JMIR Public Health and Surveillance*).

The study aimed to examine the association between smoking behavior change and the risk of heart failure (HF) among patients with type 2 diabetes. Smoking behavior changes were assessed at two consecutive health screenings between 2009 and 2012, and patients were followed up until the end of 2018 for incident HF. The authors concluded that smokers who ceased smoking were associated with lower risks of HF, whereas smokers who increased their level of smoking were associated with higher risks of HF compared to those who sustained their level of smoking. Reducing the level of smoking did not lead to a decreased risk of HF.

We applaud the authors for this valuable study but have several remarks that we feel would benefit the article’s readership. The authors state that the definition of current smokers was according to the World Health Organization; however, from the reference the authors cite, it is not clear what definition was used for the study, as the said reference [2] is concerned with thyroid cancer screening in Korea. It would be beneficial to understand the exact definition of current smokers and which tobacco and nicotine products were considered for this analysis.

Second, as the authors noted smoking behavior changes at only two consecutive health screenings, it would be interesting to understand whether such smoking behavior changes persisted during follow-up years when incident HF was detected, as this might have affected the results if only short-term smoking behavior change was reported at the second screening visit.

Third, as varenicline has been shown to potentially lower the risk of HF [3], information on smoking cessation pharmacotherapy would be helpful to assess possible bias of chosen smoking cessation pharmacotherapy, if such data were collected in the study.

Finally, the authors state that harm reduction strategy for smokers is based on reducing the number of cigarettes smoked each day. To this, we would like to add that the currently recommended harm reduction strategy includes the use of medicinally licensed nicotine-containing products [4]. Moreover, there is an ongoing discussion of alternative nicotine delivery systems used by smokers to possibly reduce the health risks of smoking [5]. Therefore, it would be useful to also collect data on the types of nicotine products used (and a relevant switch to a different nicotine product) for future studies.

We respectfully suggest considering these points, especially if a continuation of the study is planned.

## Abbreviations

HF: heart failure
